# Rationale and methods of a multicentre randomised controlled trial of the effectiveness of a Community Health Assessment Programme with Emergency Medical Services (CHAP-EMS) implemented on residents aged 55 years and older in subsidised seniors’ housing buildings in Ontario, Canada

**DOI:** 10.1136/bmjopen-2015-008110

**Published:** 2015-06-11

**Authors:** Gina Agarwal, Beatrice McDonough, Ricardo Angeles, Melissa Pirrie, Francine Marzanek, Brent McLeod, Lisa Dolovich

**Affiliations:** 1 Department of Family Medicine, McMaster University, Hamilton, Ontario, Canada; 2 Public Health Services, Hamilton, Ontario, Canada; 3 Hamilton Paramedic Services, Hamilton, Ontario, Canada; 4 Centre for Evaluation of Medicines, Hamilton, Ontario, Canada

**Keywords:** DIABETES & ENDOCRINOLOGY, GERIATRIC MEDICINE, SOCIAL MEDICINE

## Abstract

**Introduction:**

Chronic diseases and falls substantially contribute to morbidity/mortality among seniors, causing this population to frequently seek emergency medical care. Research suggests the paramedic role can be successfully expanded to include community-based health promotion and prevention. This study implements a community paramedicine programme targeting seniors in subsidised housing, a high-risk population and frequent users of emergency medical services (EMS). The aims are to reduce EMS calls, improve health outcomes and healthcare utilisation.

**Methods/analysis:**

This is a pragmatic clustered randomised control trial in four communities across Ontario, Canada. Within each, four to eight seniors’ apartment buildings will be paired and within each pair one building will be randomly assigned to receive the Community Health Assessment Programme through EMS (CHAP-EMS) intervention, while the other building receives no intervention. During the 1-year intervention, paramedics will run weekly sessions in a common area of the building, assessing risk factors for cardiovascular disease, diabetes and falls; providing health education and referrals to community programmes; and communicating results to the participant's primary physician. The primary outcomes are rate of emergency calls per 100 residents, change in blood pressure and change in Canadian Diabetes Risk (CANRISK) score, as collected by the local EMS and study databases. The secondary outcomes are change in health behaviours, measured using a preintervention and postintervention survey and healthcare utilisation, available through administrative databases. Analysis will mainly consist of descriptive statistics and generalised estimating equations, including subgroup cluster analysis.

**Ethics/dissemination:**

This study is approved by the Hamilton Integrated Research Ethics Board and will follow the Tri-Council Policy Statement. Findings will be disseminated through reports to local stakeholders, publication in peer-reviewed journals and conference presentations.

**Trial registration number:**

NCT02152891.

**Strengths and limitations of this study**

- This study will evaluate a new model of healthcare, better using the skills and training of paramedics to deliver a health assessment, prevention and promotion intervention in their community.
- A strength of this study is the randomised controlled design and the pragmatic approach to encourage applicability to real world clinical practice and policy.
- Also, this study applies an adaptation of an intervention already demonstrated to reduce hospitalisations for cardiovascular disease in other settings.
- Not every community will have subsidised housing buildings for seniors, which will limit the generalisability of our findings. However, this setting is sufficiently common that the results will be valuable to many communities.

## Introduction

Morbidity from chronic diseases and falls causes many older adults to seek emergency medical care. In 2007, chronic diseases (including cardiovascular diseases (CVDs) and diabetes) were responsible for 79% of all deaths in the Canadian province of Ontario.^1^ Similar rates are observed nationally and internationally,^2^ leading the WHO to develop a global strategy to reduce chronic disease by addressing the common risk factors (physical inactivity, unhealthy diet, tobacco use and harmful use of alcohol).^3^ Chronic diseases are strongly correlated with ageing and it is projected that by 2031, nearly 25% of Ontario residents will be 65 years or older.^4^ These diseases diminish quality of life and the potential of our communities,^1^ and, hence, it is important to strive for chronic disease prevention and management among older adults.

Elevated blood pressure (BP) is a readily preventable cause of CVD, and the WHO has identified high BP as a leading risk factor for death.^5^ In addition, hypertension and diabetes frequently coexist, which further compounds the challenges of avoiding CVD. In Canada, 63% of adults with diagnosed hypertension also had diagnosed diabetes.^6^ It is forecast that with the increasing incidence of diabetes, 3.7 million Canadians will be living with this illness by 2018–2019.^7^

In addition to CVD and diabetes, falls contribute to morbidity among older adults. It is estimated that one in three persons over the age of 65 is likely to fall at least once each year,^8^ ^9^ and every 30 min an older adult in Ontario is admitted to hospital due to a fall.^10^ Falls decrease quality of life, impact the individual's ability to continue living within the community and are the sixth most common causes of death among seniors.^10^ Most importantly, almost all falls can be avoided through proper screening, intervention and prevention.

Studies have shown that older adults account for more than a third of all Emergency Medical Service (EMS) calls related to cardiopulmonary conditions, diabetes and falls.^11–14^ This may be due to the fact that older adults with high comorbidity (>3 chronic conditions) report poorer health, take more prescription medications and have the highest rate of healthcare visits.^15^ Data from Hamilton Paramedic Service indicate that tenants in ‘seniors’ buildings’ in underserved areas of lower socioeconomic status in Hamilton, a medium sized city in Canada, with a population of just over 500 000 people, are the most frequent callers to the emergency 911 medical service. On average, each paramedic visit and subsequent mandatory hospital emergency department (ED) visit costs around $1044 Canadian dollars.^16^ ^17^ Therefore, interventions that reduce the escalation of EMS calls and ED visits could provide potential savings to the healthcare system. Current government approaches being explored include looking for programmes that reallocate existing resources to decrease the use of ED resources.

Studies expanding the role of paramedics to include health promotion and referral to appropriate health service providers have been successfully implemented in other settings. However, this research has been limited to case studies, mostly in rural Australia.^18–20^ Previous studies have shown that paramedics were seen as an underused human resource in rural and remote areas.^18^ Expanded roles included both clinical and primary care, such as community education and engagement, preventive services, treatment of minor illness (and locally endemic conditions) and promotion of lifestyle change to prevent and manage chronic disease.^18^ ^20^ A qualitative evaluation showed that these roles were acceptable to the paramedics and were appreciated by the community. Another study, conducted in the UK, evaluated expanding the roles of paramedics in responding to calls from the elderly living in urban areas;^19^ paramedics were given the additional role of referring patients to a general practitioner, district nurse and/or community social services, when needed.

In partnership with Hamilton Paramedic Services and City Housing Hamilton, our team has created a unique multifaceted intervention with health risk assessment (CVD, diabetes and falls), health education/promotion, appropriate referral and feedback to the family physician targeting the issues experienced by seniors that often lead to EMS calls. The intervention is called the Community Health Assessment Programme through E MS (CHAP-EMS) and is based on the Cardiovascular Health Awareness Programme (CHAP) model that focused on hypertension assessment^21^ and the Community Health Awareness of Diabetes (CHAD) Programme, which added screening for diabetes.^22^ ^23^ CHAP is a community-based, primary care-centred, volunteer-led, free CVD risk assessment and blood pressure (BP) monitoring programme that was demonstrated in a community cluster randomised control trial to reduce annual hospital admissions at the population level due to stroke, heart failure, and heart attacks by 9% in people aged 65 and over in Canada.^21^ CHAD was a community adaptation of CHAP in which the addition of a diabetes risk assessment tool was demonstrated to be feasible in one community, and had potential to identify individuals with diabetes earlier than through usual care.^23^ The CHAP-EMS programme adapts CHAP and CHAD by extending individual-level and population-level strategies for primary prevention and ‘closes the loop’ by linking participants to follow-up care. Unlike CHAP and CHAD, CHAP-EMS provides its sessions within a common area of the seniors’ apartment buildings, increasing accessibility for a population that may find it difficult to attend programmes in the community. Also, CVD screening in the CHAP-EMS programme includes measurement with a clinically-validated, professional automated BP machine that takes multiple measures and provides an average. Screening also includes diabetes risk assessment using the CANRISK tool,^24^ and screening for fall risk using the Timed Up and Go test.^25^ Finally, CHAP-EMS utilises existing resources such as local wellness programmes, referrals to Community Care Access Centre (CCAC) and staffing by accommodated paramedics (paramedics with personal physical limitations due to injury or pregnancy) trained in health promotion.

The CHAP-EMS pilot was conducted weekly for 1 year in one seniors’ building.^26^ During this period, 79 residents attended the sessions and 48 residents (61%) visited more than once; 40.7% were identified as having undiagnosed elevated BP, and after five visits the mean BP dropped significantly. Similarly, 66.7% of participants had moderate or high risk of developing diabetes, based on their CANRISK score; at their 6-month follow-up, 15% dropped to a lower risk category. Finally, the number of EMS calls decreased by 25% (CI 17.3 to 34.0%) compared to the 12 months preintervention.^26^ Since initial pilot testing was so promising, it is necessary to conduct a robust evaluation of the effectiveness of the CHAP-EMS Programme using randomised controlled trial methodology to understand the effectiveness of CHAP-EMS once scaled up and applied across a number of settings. The evaluation aims to answer the following research questions:

### Primary questions

- Will there be a *difference in the rate of EMS calls* in subsidised seniors’ housing buildings receiving the CHAP-EMS programmes compared to buildings not receiving the programme?
- Will there be a *change in measured systolic and diastolic BP* in senior residents living in subsidised seniors’ housing after receiving the CHAP-EMS programme compared to their own baseline measurement?
- Will there be a *difference in 10-year diabetes risk, as measured using the CANRISK tool,* in senior residents living in subsidised seniors’ housing after receiving the CHAP-EMS programme (healthy lifestyle education) compared to their own baseline measurement?

### Secondary questions

- Will there be a *difference in the health perceptions, behaviours, intentions regarding behaviours, health literacy and knowledge of resources* in senior residents living in subsidised seniors’ housing after receiving the CHAP-EMS programme compared to their own baseline measurement, and compared to seniors in a building not receiving the programme?
- *Will there be a difference in health-seeking behaviour (eg, number of hospital emergency room visits and primary care visits)* by senior residents living in subsidised seniors’ housing compared to baseline, and compared to seniors in a building not receiving the programme?

## Methods and analysis

### Study design

This study is designed as a pragmatic clustered randomised controlled trial with parallel intervention and control groups. The trial will occur in the Hamilton, York, Guelph, Sudbury and Simcoe regions of Ontario, Canada; a current list of participating regions can be found on clinicaltrials.gov under trial number NCT02152891(see online supplementary appendix A). Within each region, up to eight buildings will be selected and paired based on their characteristics. Within each pair, one building will be randomly allocated to receive the intervention and the other building will act as its control. The programme will be delivered for 1 year in the intervention buildings. Outcomes will be analysed using parallel comparisons (of intervention vs control buildings) as well as preintervention and postintervention comparisons. Process evaluation will be carried out after the 1 year study implementation and will assess the programme implementation in terms of efficiency of the processes in its delivery, participation rates, compliance and ways of improving it. All operations will be overseen by the research team at the Department of Family Medicine, McMaster University, with advice from local stakeholders (eg, public health). Local EMS will monitor the intervention session staffing on a day-to-day basis.

### Participants

Since seniors living in subsidised housing managed by community housing are typically defined as ‘senior’ if they are 55 years or older, and individuals with low socioeconomic status report poorer health^27^and increased health behaviour risk factors,^28^ CHAP-EMS has defined our study population as individuals 55 years and older residing in selected subsidised apartment buildings under the management of the local City Housing department. Only subsidised seniors’ buildings in which 60% or more of the apartment units are occupied by residents 55 years or older will be chosen. All residents 55 years and older will be included as potential participants for the individual level outcomes. Those individuals who have been staying in the buildings for less than 3 months (as visitors or guests) will be excluded. Participation in the CHAP-EMS programme will be voluntary. Names of participants will remain secure in the CHAP-EMS database and will not be disclosed to any third party or City Housing official.

### Allocation of intervention

First, the buildings will be matched into similar pairs based on geographic location, numbers of units within the building, proportion of units occupied by seniors, existing EMS call rate (number of calls/100 apartment units) in the 2 years prior to the intervention period and the types of social programmes offered to residents. Then, for each matched pair, one building will be randomised to receive the intervention and the other will not receive the intervention (control group). Block randomisation will be used with random allocation of the intervention within each block of two buildings.

Residents of the intervention buildings will have access to the CHAP-EMS programme, in additional to their usual medical care and wellness programmes, while residents of control buildings will only have access to their usual medical care and wellness programmes. Since randomisation is occurring at the building-level, it is not expected that providing usual care as the control condition will impact study recruitment, results or interpretation.

### Sample size

Given that our main outcome will be rates of calls per 100 residents per year, we calculated our sample size using the formula for the Poisson test. Based on data from the Hamilton Paramedic Services, the average rate of calls for our pilot sample was 44.06 EMS calls per year per 100 residents older than 65 years. If we assume a difference of 10% (a difference of 4.4 EMS calls per 100 residents per year) between the intervention and control groups, with 0.8 β and α of 0.05, we would need a sample of 131 per group. This is a conservative estimate of effect size since the pilot study showed a 25% reduction in EMS calls.^26^ Also, a difference of 10% is considered operationally significant by Hamilton Paramedic Services (personal communication with B McLeod). This is likely to be significant in other Ontario localities as well.

The overall sample size will be a minimum of 700 participants per arm (intervention or control) in 12 or more intervention buildings across all sites. Considering the Design Effect (cluster sampling by buildings), we can accommodate an intracluster correlation coefficient (ICC) of up to 2.5% given our proposed sample size. There is no literature regarding an ICC for a similar study.

### Recruitment process

Recruitment of intervention building residents into the CHAP-EMS programme will be organised in a multifaceted way. Existing building-based wellness groups and Tenants’ Associations will identify leaders from among the residents of the buildings who can champion the CHAP-EMS programme. The programme will also be advertised to the target population using posters in the intervention buildings. Posters will be translated into other languages as needed by the population composition in the buildings chosen.

Participants in the control buildings will receive usual care and will not be recruited for any intervention. The only contact the researchers will have with the participants of the control buildings will be to obtain survey responses regarding their health perceptions and behaviours at the beginning and end of the study. They will not be informed of the CHAP-EMS programme occurring in the matched intervention building.

### Research intervention

The intervention includes BP, diabetes and fall risk assessments, health education/promotion, targeted referral to appropriate in-house wellness sessions and community resources, identification and referral of high-risk patients to their family physician, as well as regular communication of participants’ health information to their physician. The sessions will be held weekly in a common area of the intervention apartment buildings and will be implemented by paramedics from the local paramedic service who have undergone a structured training programme (3–4 h of online, interactive training modules, including case studies and the observation of an intervention session led by another paramedic) to assure intervention fidelity.

Individual participants will voluntarily attend and will complete the informed consent process with a paramedic on their first visit (see online supplementary appendix B). Following consent, they will have their risk factors assessed and entered into a database, and they will be advised to make lifestyle changes (if applicable) with the help of available community resources and educational pamphlets. Participants with a moderate-to-high score on CANRISK will be asked to return for a fasting blood glucose capillary test, where the paramedic will guide the participant through pricking his or her own finger with a lancet and applying a drop of blood to a test strip to measure fasting blood sugar. CANRISK assessments will be repeated at 6-month intervals to assess change.

Using a prespecified algorithm, participants will be directed to appropriate services. Those identified as high risk will be immediately referred to appropriate healthcare resources, such as their primary healthcare provider. Based on a moderate risk profile and a needs assessment, participants will be referred to community resources to assist them in managing their health, specifically targeting the four common risk factors for chronic disease identified by the WHO (physical inactivity, unhealthy diet, tobacco use and harmful use of alcohol),^3^ as well as mental health and stress. These community resources are run by Community Housing and Public Health and include age-appropriate physical activities, speakers on healthy eating, social engagement opportunities with cooking demonstrations and referrals to local community resources, as required. Follow-up for identified concerns will be provided through linkages with primary healthcare providers and CCAC referrals.

Consenting participants will have their BP and risk profile sent by fax from the CHAP-EMS database to their primary healthcare provider. Participants without a family physician will be referred to CCAC for assistance with locating a suitable physician.

### Primary and secondary outcome measures

The primary outcome of EMS call rate (number of calls for building made by residents aged 55 years or older per 100 residents aged 55 years or older in the subsidised seniors’ building) will be available for each building from the EMS administrative database. The primary outcomes of change in measured systolic and diastolic BP, and difference in 10-year diabetes risk (CANRISK score), will be available from the measurements entered by the paramedics into the study database. The secondary outcomes will be difference in health perceptions, behaviours, intentions regarding behaviours, health literacy and knowledge of resources, measured by interviewer-led survey preintervention and postintervention; and difference in health-seeking behaviour (eg, number of ED visits/population 55 years and older), which will be based on administrative databases.

### Process evaluation measures

Process evaluation of CHAP-EMS will include participation rates (number of participants attending, initial attendance and repeat visits), programme delivery (eg, completion of risk assessments) and other programme evaluation measures (eg, detection rates for hypertension and diabetes).

### Data gathering procedures

Rates of EMS calls, ED visits, primary care visits and other healthcare utilisation will be collected from the administrative database of the local paramedic services, hospital ED databases, Institute for Clinical Evaluative Sciences (ICES) databases, the CHAP-EMS database and consenting participants’ primary care charts. Administrative data will be collected preintervention and postintervention, and retrospectively for the 12 months of intervention.

Health perceptions, behaviours and intentions regarding behaviours, health literacy and knowledge of resources related to chronic disease risk factors will be collected from consenting participants using an interviewer-led survey called the Health Awareness and Behaviour Tool (HABiT). This questionnaire covers the following domains: demographics, health status and quality of life, knowledge and risk factors (cardiovascular and diabetes), health utilisation and access to healthcare, and perceived concern and understanding of risk. It was developed by the research team, loosely based on existing validated questions. It has been pretested, piloted and validated in a sample of older adults in Ontario. Each item will be assigned a score and participant questionnaire results will be summarised as numerical scores. These outcomes will be analysed at the individual level and linked to administrative databases.

There will be two periods of data collection with the HABiT survey: a preintervention survey for residents of the intervention and control buildings administered up to 3 months prior to the intervention, and a postintervention survey administered up to 3 months immediately postintervention. Residents will be notified about the surveys through presentations at tenant engagement meetings, word-of-mouth and posters within the buildings. Surveys will be administered by trained college/university students and research assistants.

Physical measures (eg, BP, weight), risk assessment scores and process outcomes will be collected through the CHAP-EMS electronic database. The database is encrypted, and only accessible to the research team and the paramedics implementing the study.

Any urgent medical concerns identified or similar adverse events will be handled following protocol (eg, phoning the primary physician or EMS, depending on the level of urgency); these events will be recorded and reported to the ethics committee where appropriate.

### Data analysis

We will use an intention-to-treat analysis and generalised estimating equations (GEE) assuming a Poisson distribution and an exchangeable correlation structure within a building to evaluate healthcare utilisation, BP and risk assessment scores, as well as to identify factors contributing to these outcomes. Rate of EMS calls will be analysed at the building-level only, using GEE. We will perform pairwise comparisons of outcomes for each month during the preintervention and intervention periods. We will identify trends in the outcomes during the preintervention and postintervention observation periods. Subgroup analysis by clusters (each building) and by location will be performed. The HABiT survey data will be summarised as a numerical score representing the participants’ perceptions and behaviours regarding CVD and diabetes, and analysed with GEEs. The intracluster correlation coefficient (ICC) will be computed to determine the effect of clustering (by the building the participants reside in) on the participants’ scores. Other outcomes will be analysed using descriptive statistics.

All analyses requiring health administrative data will be performed in a two-step process. First, CHAP-EMS data with identifiers will be securely transferred to ICES for health record linkage, as per the participant consent form. Once linked, the data set will be de-identified for subsequent analysis. All analyses will be performed using SPSS V.20. Statistical significance will be set at α=0.05 and adjusted using the Bonferroni method for secondary analyses. Results will be reported as rate ratios, 95% CIs and associated p values.

Twice a month, the CHAP-EMS data will be reviewed and analysed to assess for errors in data entry, and assure fidelity to the planned intervention (eg, monitor referral patterns by EMS personnel and proper use of the screening tools). This trial did not warrant formal externally managed data monitoring, or a formal committee, due to its minimal risks. Instead, interim analysis will be conducted on the process measures to evaluate intervention recruitment and implementation, and to provide regular reports to local stakeholders (eg, EMS, public health, local housing). Collaborative research agreements with local partners (eg, housing) specify that aggregate data will be shared with these local stakeholders. Individual data will not be released without written permission from the participant.

The primary outcome (rates of EMS calls) and secondary outcomes will be analysed at the beginning of the intervention (preintervention data), and after the 12-month intervention period (postintervention data and full data analysis).

### Control of bias

For the primary outcome of EMS call rate, we will collect at least 1 year of data before the intervention starts and also use a parallel design (for pairwise analysis) to examine the possible effect of seasonality. For the HABiT, participants will not be informed about the group comparisons. Surveys will be anonymous to decrease the possibility of social desirability bias.

### Loss to follow-up

We do not expect a loss to follow-up (due to death or transfer of residence) of more than 5%, since this is a 1-year intervention study. Yearly death rates for males and females are 5.85 and 5.59 per 1000,^29^ which is unlikely to affect our study, despite the possibility of slightly higher rates due to the socioeconomic status of the study population.

## Ethics and dissemination

Following the Tri-Council Policy Statement, second edition,^30^ participation in this study is voluntary and all participants will provide written informed consent. Participants are able to withdraw at any point. Any protocol changes will be reviewed by the Hamilton Integrated Research Ethics Board and updated on the clinicaltrials.gov registry.

The study findings will be shared with the stakeholders in each community (eg, public health, community housing and emergency medical services), published in peer-reviewed journals and presented at conferences. Authorship will follow the criteria recommended by the International Committee of Medical Journal Editors.

## Discussion

This study will represent one of the first rigorous evaluations of community interventions across Ontario, within Canada and North America, using community paramedics and utilising a health promotion approach for cardiovascular risk; the study will try to ascertain changes in outcomes of lifestyle and health behaviour. Although the BP and cardiovascular screening of CHAP has been implemented and tested in small to mid-sized communities across Ontario,^21^ it has not been tested in this scenario of hard-to-reach seniors, with these outcomes, and using accommodated paramedics to assist with data collection, assessment, education and links to community resources, in the format of the CHAP-EMS programme. The strengths of this study include the use of a randomised controlled design, the adaptation and reconstitution of an intervention already demonstrated to reduce hospitalisations for cardiovascular disease, a pragmatic approach so as to generate findings regarding the effectiveness of the intervention that would be most applicable to real world clinical practice and policy, the delivery of the intervention in multiple communities each with their own context and the repositioning of healthcare resources (paramedics) to better maximise their skills and knowledge of the communities they serve. Applying the Precis-2 score,^31^ which is a measure of the pragmatic nature of a trial in a real-world setting, of 9 domains and a maximum score of 45, the CHAP-EMS protocol scored 41, indicating a pragmatic and useful approach. In more detail, the Precis-2 score considers the following elements as they are applied in the CHAP-EMS RCT as follows: eligibility—participants are those inhabiting seniors’ buildings and will be part of usual care (score 5); recruitment—not much extra effort is made to recruit participants over and above that which would be used in the usual care setting (score 5); setting—a very pragmatic choice of setting is utilised (score 5); organisation—resources utilised are those of the EMS service, and the organisation of care delivery in the intervention arm needs coordination (score 3); flexibility (delivery)—a fairly pragmatic choice with slight flexibility to usual care (score 3); flexibility (adherence)—a very pragmatic choice involving no more than usual encouragement to adhere to the intervention (score 5); follow-up—no more than usual follow-up (score 5); primary outcome—the outcome is of obvious importance to participants (score 5); and primary analysis—using intention to treat with all available data (score 5).

One limitation of our approach is that not every location will have subsidised housing buildings containing a majority of seniors, which will limit the generalisability of our findings. However, in Canada, this setting is sufficiently common, and that is critical in testing this strategy. Also, the study is focused on more urban areas with a large population of seniors living within a certain locale, and therefore the results will be limited to similar settings.

With our team's experience in implementing and evaluating the CHAP trial, we believe that expanding the programme to serve seniors living in subsidised housing, adding diabetes and falls screening and utilising accommodated paramedic personnel to run the programme, will be effective in lowering rates of EMS calls and ED visits through increasing risk factor detection and awareness, health education and referral to appropriate services. The relative decrease in EMS calls and ED visits will have implications in terms of healthcare savings. At the current time, real findings of this nature are not available and we need more research to understand what is clinically and policy-wise relevant. We also believe that the programme can improve the health behaviour of building occupants, which is expected to improve health outcomes. Such findings will have significant policy implications in favour of its widespread implementation. This study is in keeping with Canada's *Living Longer, Living Well* strategy^32^ for utilising allied health professionals more appropriately and extending their roles within health promotion and disease prevention. It addresses the question of the effectiveness of a novel form of health services delivery on healthcare processes, health outcomes and health resource utilisation.
